# Effects of thermal treatment on the properties of defatted soya bean flour and its adhesion to plywood

**DOI:** 10.1098/rsos.180015

**Published:** 2018-05-02

**Authors:** Bing-Han Zhang, Bo Fan, Ming Li, Yue-Hong Zhang, Zhen-Hua Gao

**Affiliations:** College of Material Science and Engineering, Northeast Forestry University, 26 Hexing Road, Harbin 150040, People's Republic of China

**Keywords:** wood adhesive, thermal treatment, defatted soya bean flour, water resistance

## Abstract

With an attempt to economically and efficiently improve the water resistance of defatted soya bean flour (DSF)-based wood adhesives, DSF was subjected to thermal treatment at various temperatures (65°C, 80°C, 95°C, 110°C and 125°C) for 30 min. The effects of thermal treatment temperature onto the chemical structure, crystalline degree, water-insoluble content and acetaldehyde value of the thermally treated DSF (T-DSF) were investigated. The thermal stabilities and bonding properties of soya bean adhesives prepared from T-DSF and cross-linker epichlorohydrin-modified polyamide (EMPA) were also investigated. Test results indicated that both the water-insoluble content and the acetaldehyde value of T-DSF increased after thermal treatment, reaching the highest values of 27.28% and 26.81 mg g^−1^, respectively. All plywood bonded with the T-DSF-based adhesive withstood a 28 h boiling–dry–boiling accelerated ageing treatment, while plywood bonded with the DSF-based adhesive delaminated after 4 h of water boiling, demonstrating the significantly improved water resistance of the T-DSF-based adhesives. Related analyses also confirmed that this improvement was due to: (i) the formation of insoluble cross-linked structures of T-DSF resulting from protein–protein self-cross-linking reactions and the protein–carbohydrate Maillard reaction and (ii) increased cross-linking efficiency between T-DSF and cross-linker EMPA owing to more T-DSF-reactive groups being released after thermal treatment.

## 1. Introduction

Formaldehyde-based adhesives are widely used in the timber industry [1]. However, these adhesives are mainly derived from non-renewable petroleum resources. Furthermore, formaldehyde is considered a hazardous air pollutant, volatile organic compound and reasonably anticipated human carcinogen. Therefore, there is an urgent need to develop environmentally safe wood adhesives employing renewable and sustainable resources. Among various renewable resources, vegetable protein is considered a very promising raw material for preparing wood adhesives [2,3].

Soya bean is widely cultivated globally and mainly used as a source of edible oil, with defatted soya bean flour (DSF) afforded as a by-product. DSF is mainly used as an animal feed with a low added value. Soya bean protein isolate, extracted from DSF, is widely used to formulate wood adhesives due to its high protein content (greater than 90%). However, the price of soya bean protein isolate is three to five times higher than that of DSF, making it less attractive as a starting material for preparing wood adhesives. Furthermore, DSF is among the most promising candidates for preparing wood adhesives owing to its advantages [4,5]. Generally, DSF contains approximately 50% soya bean protein, 40% soya bean carbohydrate and 10% minor components [6]. Among them, soya bean protein acts as the major adhesion component in DSF-based adhesives because it has a wide range of reactive functional groups, such as –NH_2_, –COOH, –OH and –SH, and has the ability to disperse in solution and interact with wood. However, soya bean carbohydrate component has been confirmed to have a negative effect on the water resistance of DSF-based adhesives due to its abundant hydroxyl groups with high hydrophilicity [7–11]. Therefore, DSF-based adhesives exhibit poor water resistance and cannot withstand water boiling tests. Many attempts have been made to improve the water resistance of DSF-based adhesives to meet commercial application requirements.

Currently, DSF modification is mainly focused on its soya bean protein components. Native soya bean protein has a highly compact globular structure. This unique globular structure prevents protein from effectively and sufficiently absorbing or interacting with the wood substrate to form good interfacial adhesion. Therefore, the first steps generally reported use physical, chemical and biological methods to unfold the protein structure and expose more reactive groups, including heat treatment [12], acid [13], alkali [14], salt [15], urea [16], sodium dodecyl sulfate [17] and sodium bisulfite [18]. This is followed by cross-linking or grafting modification with polyamidoamine [19], glyoxal-polyisocyanate [13], maleic anhydride [19], melamine–urea–formaldehyde resins [20], phenol–formaldehyde resins [21] and polyamidoamine epichlorohydrin resins to further improve the water resistance of the DSF-based adhesive [22,23]. However, these DSF-based adhesives still have poor water resistance and can only withstand the test by soaking in the 63°C water for 3 h. This is mainly attributed to the soya bean carbohydrate component within DSF not being sufficiently modified to form insoluble cross-linking structure.

Therefore, making full use of the soya bean carbohydrate component within DSF and turning it into an insoluble structure are important for further improving the water resistance of DSF-based adhesives [7]. Reports have described using Viscozyme L to hydrolyse the soya bean carbohydrate within DSF to reducing sugars, with the resultant reducing sugars then able to self-cross-link with the soya bean protein component to prepare a DSF-based adhesive with improved water resistance [24]. However, the high cost and high specificity of the enzymes have greatly hindered the commercial application of this DSF-based adhesive. Therefore, a more facile and affordable route to modify DSF is required.

The heat treatment of soya bean protein isolate has been widely investigated in past decades, and the effects of thermal treatment on the structural characteristics of soya bean protein have been reported [25]. It has been demonstrated that thermal treatment leads to the dissociation of proteins into subunits, and unfolds the quaternary, tertiary and secondary structures of soya bean protein. This exposes hydrophobic groups buried within the globular structure [26], releases a large number of reactive groups and provides more reactive sites, which not only increase absorption interactions via hydrogen bonding between the proteins and wood surface, but also facilitate possible sulfhydryl(S–H)–disulfide(S–S) interchange in the subunit [27,28]. Moreover, thermal treatment of soya bean protein isolate is also accompanied by a decrease in solubility, resulting from the self-cross-linking or self-aggregation of soya bean protein.

However, limited information is available on analysing the effect of thermal treatment on the structures and properties of DSF. The interaction between soya bean protein component and soya bean carbohydrate component within DSF during thermal treatment is of great interest, because carbohydrates have the potential to react with soya bean protein via a Maillard reaction to form a self-cross-linked network structure [7,29] and thus can effectively improve the water resistance of DSF.

In this study, the effects of thermal treatment on the structures and properties of DSF were evaluated, and the self-cross-linking reaction of soya bean proteins–soya bean protein and the Maillard reaction between soya bean protein and soya bean carbohydrate of thermally treated DSF (T-DSF) were confirmed. Furthermore, T-DSF was blended with a cross-linker epichlorohydrin-modified polyamide (EMPA) to prepare adhesives for plywood, and their bond strength and water resistance were investigated in detail by plywood evaluation.

## 2. Material and methods

### 2.1. Materials

DSF with an average protein content of 53.4% (particle size, less than 0.096 mm) was purchased from Harbin High Tech Soybean Food Co. Ltd, China. EMPA with a solid content of 13.8%, pH of 2.6 and viscosity of 96.4 mPa s (25°C) was synthesized in our laboratory using diethylenetriamine, adipic acid and epichlorohydrin. Birch veneers with dimensions of 420 × 420 × 1.6 mm and moisture contents of 6–8% were supplied by a local plywood plant.

### 2.2. Thermal treatment of defatted soya bean flour

DSF was first put on an open metal plate to form a thin layer and then the plate containing DSF was placed in an oven at various temperatures (65°C, 80°C, 95°C, 110°C and 125°C) for 30 min. T-DSF was then ground into fine powder, passed through a 160-mesh sieve and labelled T-DSF-X (where X represents the thermal treatment temperature). Native DSF powder without thermal treatment was also passed through a 160-mesh sieve, selected as control and labelled as DSF.

### 2.3. Fourier transform infrared spectrum analysis

Fourier transform infrared (FT-IR) spectra of the dried T-DSF and DSF samples were recorded in the wavenumber range of 450–4000 cm^–1^ on a Spectrum One FT-IR spectrophotometer (Nicolet Co., USA). A total of 32 scans were performed at a resolution of 4 cm^−1^. Peak decomposition was performed in the region of 1700–1600 cm^–1^, which was assigned to the amide I band of soya bean protein. A second-derivative analysis was conducted using the following procedure to obtain quantitative information about the secondary structures of the protein in DSF: after baseline correction, Fourier self-deconvolution and deconvoluted (differential) spectra were resolved and then individual component bands were quantified according to a Gaussian curve fit (GCF). The procedure maintained the initial band positions with an interval of 4 ± 1 cm^–1^, excluding bands with negative heights, and with the bandwidth kept within the expected limits, in agreement with theoretical boundaries or predictions. The relative amounts of the various secondary structures of DSF were determined from the second derivative of amide I using the areas under bands assigned to a particular substructure. The difference between the measured spectrum and the curve fit was calculated as an internal control of the success of the curve-fitting process.

### 2.4. X-ray diffraction spectroscopy analysis

The change in crystalline structure of native DSF and T-DSF samples at various temperatures was recorded on a *D*/max-2200 diffractometer (Rigaku, Tokyo, Japan) using a Cu-K*α* source, and diffraction data were collected from 5° to 50° with a step interval of 0.02°, an accelerating voltage of 40 kV and a current of 30 mA.

### 2.5. Thermogravimetric analysis

Thermogravimetric analysis (TGA) was performed on a TA Instruments Discovery TGA. All samples were tested from room temperature to 800°C at a scanning rate of 10°C min^−1^ with a nitrogen purge of 25 ml min^−1^.

### 2.6. X-ray photoelectron spectroscopy tests

The surface chemical composition of native DSF and T-DSF samples was analysed on a K*α* X-ray photoelectron spectrometer (Thermo Fisher Scientific, USA) with monochromatic Al-K*α* radiation at 100 W. High-energy photoemission spectra were recorded using a pass energy of 50 eV and resolution of 0.1 eV.

### 2.7. Water-insoluble content determination of defatted soya bean flour and thermally treated defatted soya bean flour

To a 250 ml flask equipped with mechanical stirring and reflux equipment, approximately 2.0 g of the samples (DSF and T-DSF, passed through a 160-mesh sieve, *W*_0_, accurate to 0.0001 g) and distilled water (200.0 g) were added. The mixture was then stirred under reflux for 4 h. The dispersion was subsequently cooled to room temperature, filtered and the resultant residue was rinsed twice with distilled water (50 ml). The residue on the glass filter paper (*W*_1_, accurate to 0.0001 g) was oven-dried at 120°C to a constant weight (*W*_2_, accurate to 0.0001 g). The boiling water-insoluble content was defined as the mass percentage of soya bean protein insoluble in boiling water and calculated using [(*W*_2 _− *W*_1_)/*W*_0_] × 100%.

### 2.8. Acetaldehyde value of defatted soya bean flour and thermally treated defatted soya bean flour

A 100 ml reaction flask equipped with a mechanical stirrer, thermostat and reflux condenser was charged with approximately 1.5 g of samples (DSF and T-DSF) with a particle size of over 160 mesh (*m*_1_, accurate to 0.0001 g), water (50.0 ml) and 40 wt% acetaldehyde (5.0 ml). The pH of the mixture was then adjusted to 8.5–8.7 using a 20 wt% sodium hydroxide solution. The mixture was then maintained at 50 ± 2°C for 120 min. The reaction mixture was allowed to cool to room temperature and then diluted to 1000 ml in a volumetric flask. The unreacted acetaldehyde content in the diluted filtrate (*F*_1_, mmol l^−1^) was tested according to the method described in [30]. Three blank tests were conducted under the same conditions without DSF to determine the total free acetaldehyde content (*F*_0_, mmol l^−1^) prior to reaction. The acetaldehyde value (mg g^−1^) of the sample was defined as the equivalent mass of acetaldehyde (mg) that can react with 1 g of solid defatted soya bean flour and was calculated using (*F*_0 _− *F*_1_) × 44 *m*_1_.

### 2.9. Preparation of defatted soya bean flour-based adhesives

The DSF-based adhesives were prepared by mechanically blending a mixture of T-DSF (35 parts) and liquid EMPA solution (100 parts, by mass) at room temperature until no soya bean flour particle clusters were observed. The adhesive prepared from DSF and EMPA was used as control and labelled as DSF + EMPA. The solid content of all adhesives was adjusted to 35.5 wt% in water before use.

### 2.10. Bond strength and water resistance by plywood evaluation

Three-ply plywood panels were fabricated with a liquid adhesive loading of 180 g m^−2^ (single glue line) by coating adhesive onto both faces of the core veneer. The adhesive-coated veneer was cold-pressed at 1.1 MPa at room temperature for 45 min and then hot-pressed at 120°C and 1.3 MPa for 4 min. After hot pressing, the panels were stored under ambient conditions for at least 24 h before testing. A total of 40 specimens with a glued area of 25 mm × 25 mm were cut according to commercial standard JIS K6806-2003 to determine the bond strength (20 specimens) and water resistance (or bond durability, 20 specimens) in terms of the dry strength and aged wet strength, respectively, using a tensile testing machine with a crosshead speed of 5 mm min^−1^. Specimens for aged wet strength testing underwent a 28 h boiling–dry–boiling treatment (4 h of boiling, 20 h of oven-drying at 63 ± 2°C and 4 h of boiling) and were then cooled to room temperature by soaking in ambient water, and finally measured in a wet state at room temperature. By contrast, specimens for dry bond strength testing were measured in a dry state.

## 3. Results and discussion

**Figure 1.**
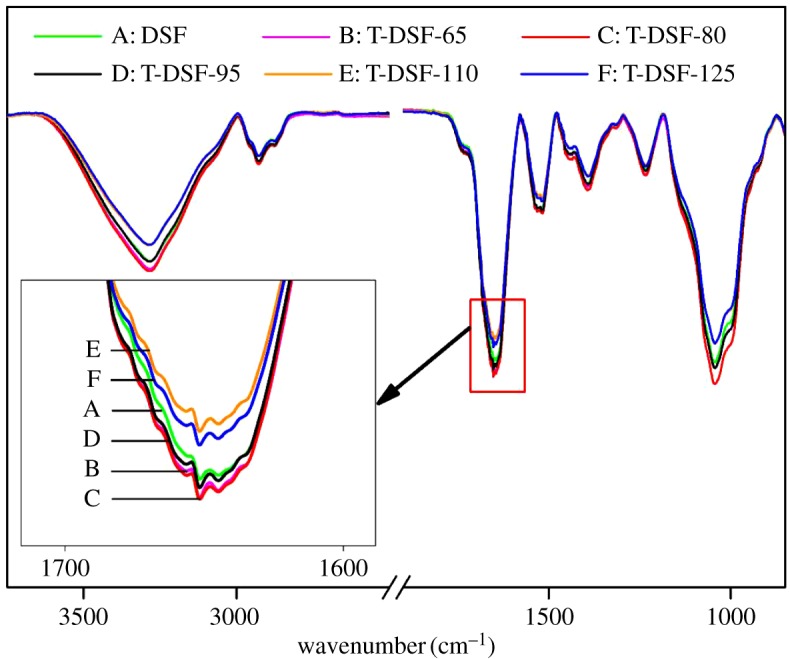
FT-IR spectra of DSF and T-DSF treated at various temperatures.

The chemical structures of soya bean flours after thermal treatment at various temperatures were performed using FT-IR (figure 1). All samples had typical infrared absorption characteristics of proteins for amide I (1651 cm^–1^, C=O stretching), amide II (1570 cm^–1^, N–H bending) and amide III (1401 and 1242 cm^–1^, C–N stretching and N–H deformation) [31]. No new absorption peak was detected for all T-DSF samples treated at various temperatures, indicating no apparent change in the chemical composition after thermal treatment of DSF.

**Figure 2.**
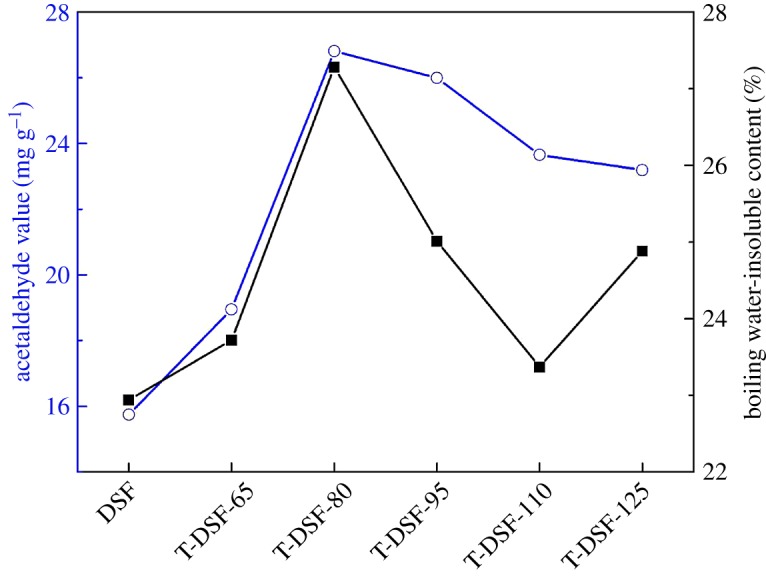
Acetaldehyde values and boiling water-insoluble contents of DSF and T-DSF.

Compared with native DSF without thermal treatment, the absorption peak intensities of T-DSF at 3281 cm^–1^ (O–H and N–H stretching vibrations), 1651 cm^–1^ (C=O stretching vibrations) and 1045 cm^–1^ (C–O stretching vibrations) were initially enhanced and then decreased, with the highest intensities produced at a treatment temperature of 80°C. This indicated that the reactive functional groups of T-DSF had reached a maximum at approximately 80°C as the thermal treatment temperature was increased from 65°C to 125°C. This result was in agreement with the acetaldehyde-value test of T-DSF, shown in figure 2, because consumption of acetaldehyde groups was attributed to the reaction between acetaldehyde and the amino groups of soya bean protein. Therefore, the acetaldehyde value represented the quantity of active amino groups in T-DSF. The acetaldehyde values of T-DSF were all higher than those of native DSF (figure 2), indicating that T-DSF contained more reactive amino groups than that of DSF, further confirming that the globular structure of the protein within DSF was gradually unfolded upon heating at 80°C and buried reactive functional groups (such as sulfydryl, amino and hydrophobic groups) were exposed [32]. With a further increase in DSF thermal treatment temperature above 80°C, new intra- and intermolecular bonds (such as hydrogen bonds, hydrophobic interactions, van der Waals forces and repolymerization of –SH and S–S via interchange reactions) reported to form insoluble aggregates [33], implying the self-cross-linking or reaggregation of soya bean protein, as confirmed by the decreasing absorption peak intensity of amino groups (figure 1) and acetaldehyde value (figure 2) in the case of T-DSF-125.

**Figure 3.**
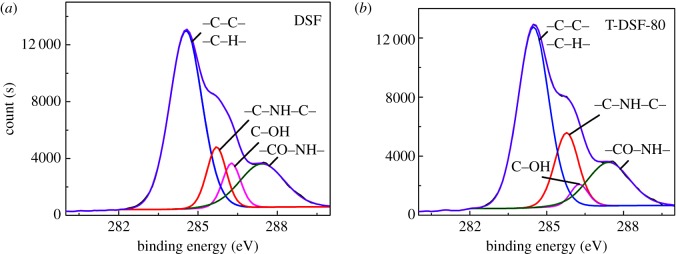
XPS spectra of DSF (*a*) and T-DSF-80 (*b*) deconvoluted into multiple sub-peaks.

Moreover, the Maillard reaction within DSF related to the range of complex reactions between amino groups of the protein and carbonyl groups (such as aldehyde groups) of the carbohydrate upon heating was further investigated by X-ray photoelectron spectroscopy (XPS) as shown in figure 3. The Maillard reaction is generally accompanied by the consumption of amino groups and the formation of Amadori compounds (–C–O–), Schiff bases (–C=N–) and pyrazines (–C–N–). However, all of these structures are difficult to observe using FT-IR (figure 1) due to peaks overlap in the fingerprint. As shown in figure 3, four C 1s photoelectron peaks were decomposed according to binding energies of carbon bonds in DSF and T-DSF, namely C1 (corresponding to C–C or C–H bonds) at 284.6 eV, C2 (corresponding to C–N–C) at 285.7 eV, C3 (related to C–OH and C–NH_2_) at 286.4 eV and C4 (assigned to –CO–NH–) at 287.8 eV.

After thermal treatment of DSF, the area of C2 increased, which was attributed to the formation of Schiff bases (–C=N–) and pyrazines (–C–N–) via reactions between the carbohydrate and protein, in agreement with previous reports [34,35]. Furthermore, the increase in area for C4 indicated the formation of more –CO–NH– moieties via condensation reactions between primary amines and carboxyl groups. The decrease in C3 indicated the consumption of amino groups in the above protein reactions. These facts confirmed that the Maillard reaction occurred between the soya bean protein component and carbohydrate component during the thermal treatment of DSF, because the globular structure of the protein was destroyed, and more reactive groups were exposed, facilitating the contact of carbonyl and amino groups. The Maillard reaction turned some soluble proteins and carbohydrates into insoluble cross-linked resultants, further improving the water resistance of T-DSF (figure 2) and its derived adhesives (figure 7).

Most of the soya bean proteins and soya bean carbohydrates components within DSF are soluble in boiling water. Therefore, DSF had the lowest water-insoluble content of 22.9% (figure 2). After thermal treatment of DSF, the water-insoluble content of T-DSF initially increased and then decreased with increasing treatment temperature from 65°C to 110°C, reaching the highest water-insoluble content of 27.3% for T-DSF-80. Further increasing the temperature above 110°C, the water-insoluble content of T-DSF continued to increase. This was mainly attributed to the thermal treatment of DSF below 80°C, causing the protein structure to unfold and expose more hydrophobic groups, while increasing the temperature from 80°C to 110°C caused the exposed reactive groups of soya bean protein to further form soluble aggregates [33]. A further increase in temperature above 110°C resulted in self-cross-linking or reaggregation of soya bean protein and the Maillard reactions between soya bean protein and soya bean carbohydrate (as confirmed by FT-IR analysis and XPS results), resulting in an increased water-insoluble content (24.9%).

**Figure 4.**
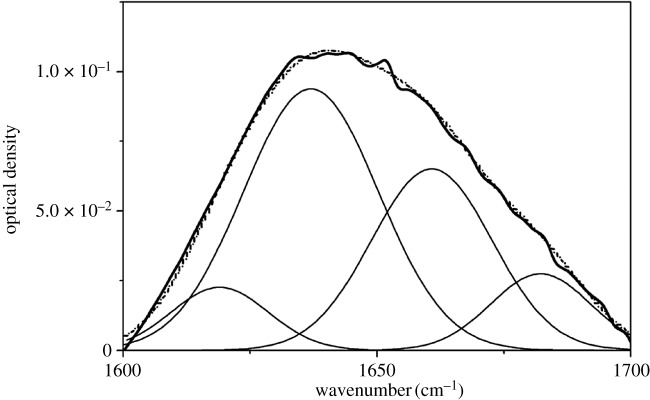
Deconvolution of amide I spectra (continuous curve), GCF bands thereof (point line) and the second-derivative spectra of DSF.

**Figure 5.**
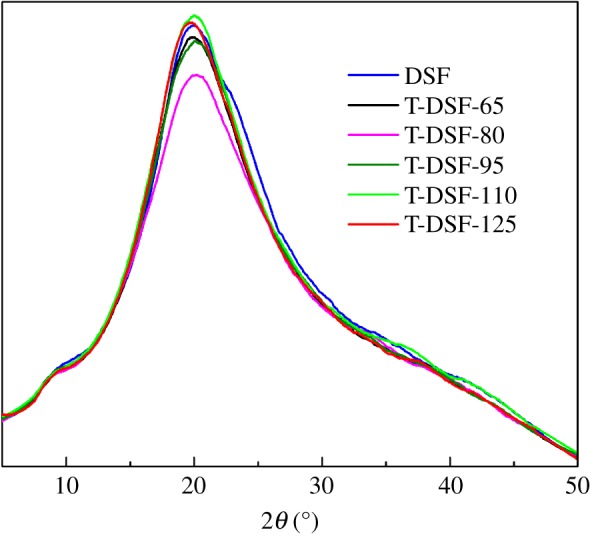
XRD patterns of DSF and T-DSF treated at different temperatures.

The deconvolution of the amide I spectra (C=O stretching vibration) in figure 4 showed variations in the secondary structures when DSF underwent thermal treatment at various temperatures. The corresponding relationships between each sub-peak and secondary structures of the soya bean protein are as follows: bands at 1618–1640 cm^–1^ and 1670–1690 cm^–1^ corresponding to β-sheet structure; 1660–1700 cm^–1^ to β-turn structure, 1640–1650 cm^–1^ to random coil structure and 1650–1660 cm^–1^ to α-helix structure [36,37]. The α-helix, β-turn, β-sheet and unordered structure contents in DSF and T-DSF are summarized in table 1. Native DSF contained α-helix, β-sheet and unordered structure contents of 36.6%, 4.0% and 45.7%, respectively. After thermal treatment at 80°C (T-DSF-80), the α-helix structure content decreased to 14.6%, while the β-sheet and unordered structure contents increased to 19.9% and 60.5%, respectively. Test results in table 1 indicated that the thermal treatment of DSF below 80°C induced the α-helix structure to form β-sheet and unordered structures, leading to a decreased crystallinity (2*θ *≈ 20) for T-DSF-80, as shown in figure 4. Further increasing the processing temperature from 80°C to 125°C, the α-helix structure content showed an increase from 14.6% to 21.9%, while the unordered structure content decreased from 60.5% to 49.0%. In other words, the ordered structure content was increased, in agreement with the X-ray diffraction spectroscopy (XRD) results (figure 5). This was mainly attributed to thermal treatment of DSF below 80°C unfolding the regular globular structure of DSF, which caused buried functional groups to be exposed, while the secondary bond that maintained the protein molecular space conformation was destroyed [36], leading to decreased crystallinity resulting from the decrease in α-helix structure and increase in unordered structure. Conversely, a further increase in thermal treatment temperature above 80°C led to a decrease in unordered structure because the unfolded T-DSF molecule would again rearrange or reaggregate to the ordered structure.

**Table 1.** Secondary structure contents in DSF and T-DSF.

samples	β-sheet (%)	unordered (%)	α-helix (%)	β-turn (%)
DSF	4.0	45.7	36.6	13.7
T-DSF-65	3.1	46.1	39.3	11.8
T-DSF-80	19.9	60.5	14.6	5.0
T-DSF-95	8.5	49.9	31.1	10.5
T-DSF-110	15.6	48.1	26.9	9.4
T-DSF-125	20.2	49.0	21.9	8.9

**Figure 6.**
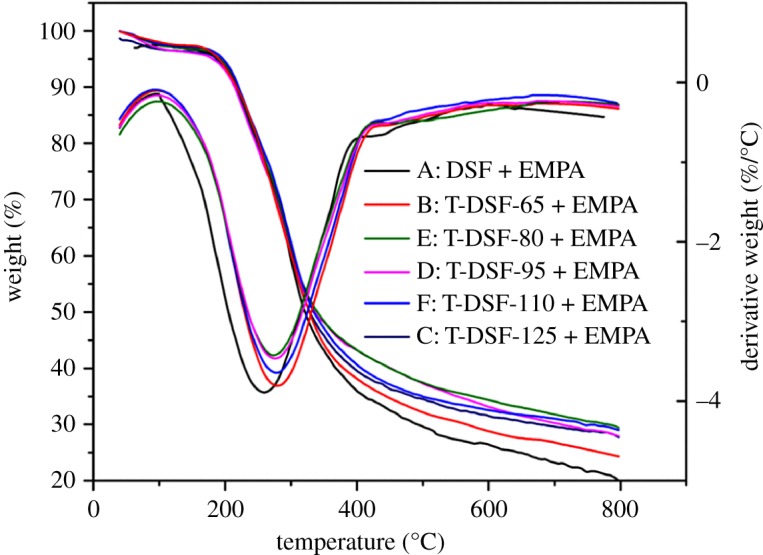
TGA curves and their derivative curves for cured DSF- and T-DSF-based adhesives.

For cured DSF- and T-DSF-based adhesives, the thermal stabilities are shown in figure 6. After thermal treatment of DSF, the thermal stabilities of T-DSF-based adhesives were higher than those of DSF-based adhesives, this was because the thermal treatment of DSF could facilitate cross-linking between EMPA and DSF, as thermal treatment resulted in self-cross-linking or reaggregation of soya bean protein and the Maillard reactions between soya bean protein and soya bean carbohydrate, as confirmed by FT-IR analysis and XPS results. Moreover, the thermal stability of T-DSF-80 + EMPA adhesive is the highest among all the T-DSF + EMPA-based adhesives. This was attributed to the increased cross-linking density because T-DSF-80 contained more reactive groups to undergo cross-linking reaction, as confirmed by FT-IR analysis and the acetaldehyde value.

**Figure 7.**
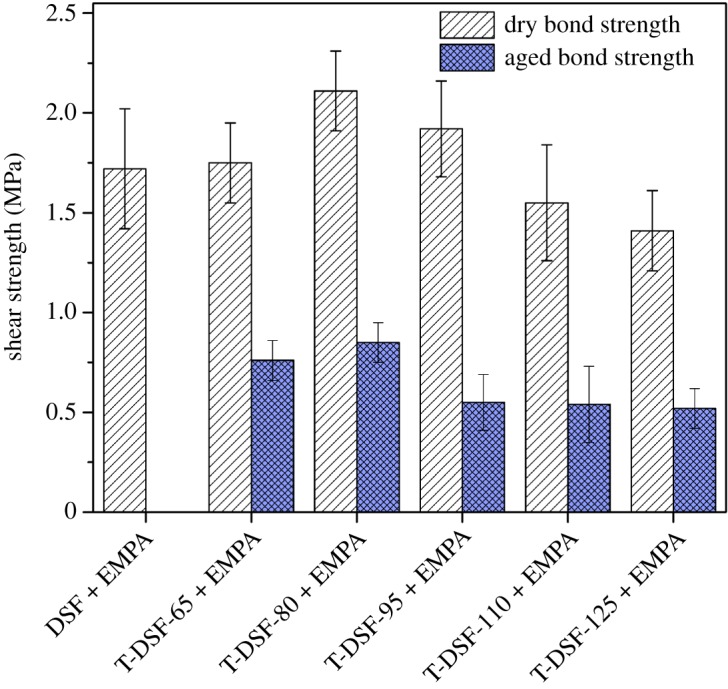
Shear strength of plywood prepared with DSF and T-DSF-based adhesives.

Plywood evaluations with adhesives formulated from DSF and T-DSF are shown in figure 7. Plywood prepared with adhesive DSF + EMPA had no aged bond strength (all test specimens delaminated within the first 4 h of water boiling), indicating poor water resistance of this adhesive because large amounts of soluble protein and carbohydrates were available within native DSF. The dissolution of the soluble protein and carbohydrates in boiling water led to the destruction of the glue line. After thermal treatment, all plywood bonded with T-DSF + EMPA adhesives could withstand a 28 h of boiling–dry–boiling aged test, and exhibited an aged bond strength of 0.52–0.85 MPa. This confirmed that thermal treatment could effectively improve the water resistance of DSF-based wood adhesive. Carbohydrate is the key negative component decreasing the water resistance of DSF adhesives [7,38]. The above improvement in water resistance was mainly attributed to protein–carbohydrate Maillard reaction during thermal treatment, which converted the soluble carbohydrate into an insoluble three-dimensional network structure.

**Figure 8.**
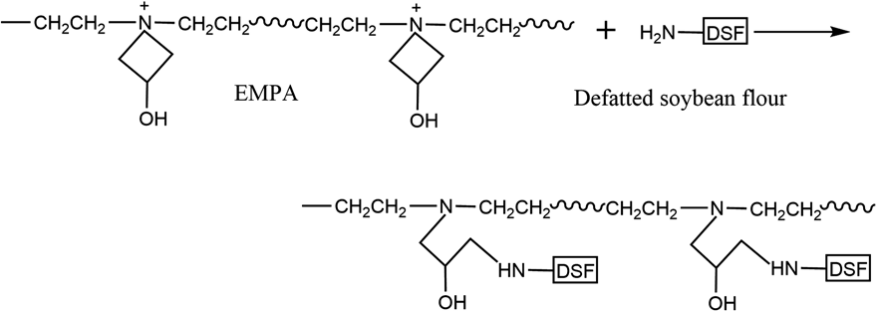
Schematic illustration of the cross-linking reaction between DSF and EMPA.

The increased acetaldehyde values of T-DSF compared with the control DSF in figure 2 indicated that some free amino groups (–NH_2_) were released during thermal treatment, which could lead to increased cross-linking efficiency. It has been confirmed that the azetidinium group of EMPA could effectively react with primary and secondary amines in soya bean protein to form three-dimensional cross-linking networks [24,39], as shown in figure 8. The corresponding tendencies of aged bond strength (figure 7) and acetaldehyde value (figure 2) at various treatment temperatures indicated that the improvement in water resistance was correlated to the released amino groups after thermal treatment, which further increased the cross-linking density. In other words, the thermal treatment of DSF could facilitate cross-linking between EMPA and DSF. However, the quantitative contributions of protein–carbohydrate Maillard reaction, protein–protein self-cross-linking, protein–EMPA cross-linking and their effects (interaction mechanisms) on the water resistance of T-DSF-based adhesive remain uncertain.

Among T-DSF samples, DSF thermally treated at 80°C had the most unordered structure, as shown in table 1, or the lowest crystalline content, as shown in figure 5. This implied that reagents, such as cross-linker and acetaldehyde, could more easily penetrate the T-DSF-80 particles and, therefore, result in higher acetaldehyde values (figure 2) and better cross-linking efficiency using cross-linker EMPA. In addition, the best boiling water-insoluble content resulting from protein–protein self-cross-linking and protein–carbohydrate cross-linking, plywood bonded with T-DSF-80 + EMPA adhesives exhibited the best water resistance, with an aged bond strength of 0.85 MPa, which was close to the required value for structural use (0.98 MPa) according to the commercial standard JIS K6806-2003. Overall, neither chemicals nor complicated equipment were required, and the soya bean carbohydrate component within DSF has been fully exploited to improve the water resistance. Therefore, the thermal treatment of DSF is an economical and effective approach to improve the water resistance of soya bean flour adhesive for wood.

## 4. Conclusion

From this study, it is clearly evident that the thermal treatment of DSF effectively improved the water resistance of DSF-based adhesive. These adhesives were able to withstand a 28 h boiling–dry–boiling ageing test and exhibited an aged bond strength in the range of 0.52–0.85 MPa. The optimal thermal treatment temperature was 80°C. Thermal treatment can facilitate protein–protein self-cross-linking, protein–carbohydrate Maillard reactions and protein–EMPA cross-linking because it unfolds the globular structure of soya bean protein and releases buried functional groups. However, the quantitative contributions of protein–carbohydrate Maillard reactions, protein–protein self-cross-linking and protein–EMPA cross-linking, and their effects on the water resistance of T-DSF-based adhesives remain unclear.

## Data accessibility

All original data are deposited at the Dryad Digital Repository: (http://dx.doi.org/10.5061/dryad.78vr884) [40].
